# Mycobacterial signaling through toll-like receptors

**DOI:** 10.3389/fcimb.2012.00145

**Published:** 2012-11-23

**Authors:** Joyoti Basu, Dong-Min Shin, Eun-Kyeong Jo

**Affiliations:** ^1^Department of Chemistry, Bose InstituteKolkata, India; ^2^Department of Microbiology and Infection Signaling Network Research Center, Chungnam National University School of MedicineDaejeon, South Korea

**Keywords:** mycobacteria, vitamin D, autophagy, antimicrobial peptides, innate immunity

## Abstract

Studies over the past decade have helped to decipher molecular networks dependent on Toll-like receptor (TLR) signaling, in mycobacteria-infected macrophages. Stimulation of TLRs by mycobacteria and their antigenic components rapidly induces intracellular signaling cascades involved in the activation of nuclear factor-κB and mitogen-activated protein kinase pathways, which play important roles in orchestrating proinflammatory responses and innate defense through generation of a variety of antimicrobial effector molecules. Recent studies have provided evidence that mycobacterial TLR-signaling cross talks with other intracellular antimicrobial innate pathways, the autophagy process and functional vitamin D receptor (VDR) signaling. In this article we describe recent advances in the recognition, responses, and regulation of mycobacterial signaling through TLRs.

## Introduction

Tuberculosis remains a serious health problem worldwide, causing an alarming two million deaths annually (WHO, 2010). One-third of the global population is latently infected with *Mycobacterium tuberculosis* (Mtb). In this state, healthy immune-competent individuals are able to combat infection by mounting of an effective immune response (Huynh et al., 2011). When the immune system is compromised, the bacteria are capable of replicating, disseminating, and causing a progressive infectious disease (Huynh et al., 2011). Thus, appropriate and efficient mounting of host immune responses are required for controlling tuberculosis infection. Protective immunity to mycobacteria mainly requires an interplay between innate immune responses and Th1-dependent cellular immune responses, which cooperatively contribute to clear the major human pathogen, Mtb (Zuñiga et al., 2012).

During infection of macrophages, Mtb and its components encounters innate immunity, which operates through a variety of germline-encoded pattern recognition receptors including Toll-like receptors (TLRs) for recognition of various molecular patterns of mycobacteria (Jo et al., 2007; Natarajan et al., 2011). The innate immune response triggered by engagement of TLRs, involves recruitment of cytoplasmic adaptor proteins and intracellular signaling molecules resulting in phagosome maturation and the synthesis of pro-inflammatory cytokines (Sundaramurthy and Pieters, 2007; Natarajan et al., 2011). Antimicrobial responses through TLR-dependent triggering of intracellular signaling cascades, involves the activation of nuclear factor (NF)-κB in murine macrophages and vitamin D receptor (VDR) signaling in human monocytes/macrophages (Sundaramurthy and Pieters, 2007; Jo, 2010; Yuk et al., 2012). Recent studies have also highlighted that the autophagy pathway is critically involved in degradation/elimination of intracellular microbes, including Mtb (Deretic, 2012; Mintern and Villadangos, 2012). Pathogenic mycobacteria utilize a unique strategy to survive within macrophages. They have developed tactics to escape from host autophagic process. They also expose multiple ligands that are recognized by host TLRs. An understanding of the complex mechanisms involved in this hide-and-seek game (host-pathogen interaction) is required for development of novel intervention strategies to combat tuberculosis.

In this Review, we focus on emerging data showing that mycobacteria and their ligands activate or modulate TLR signaling pathways. Specifically, we discuss the mechanisms by which TLR signaling regulates the initiation and progression of innate immune responses, and eradication of intracellular mycobacteria through cooperation with autophagy and VDR signaling pathway.

## Overview of TLRs in mycobacterial infection

Mtb primarily invades the lung alveoli via inhalation and alveolar macrophages are the principal cell types infected by this pathogen (Huynh et al., 2011). When Mtb are internalized by alveolar macrophages or other innate cells, they encounter a variety of intracellular innate receptors including TLRs. So far, there is evidence that mycobacterial components are engaged by TLR2 in association with TLR1/TLR6, by TLR4, or by TLR9 (which recognizes Mtb DNA) (Doherty and Arditi, 2004; Jo et al., 2007). Earlier *in vivo* studies showed somewhat discordant results in susceptibility of mice deficient in several TLR-related genes, including TLR2, TLR4, TLR6, or MyD88, in Mtb infection (Doherty and Arditi, 2004; Quesniaux et al., 2004). It was also reported that TLR2/4/9-deficient mice have no apparent difference in activation of antigen-specific T cells, and production of pro-inflammatory cytokines and interferon (IFN)-γ, when compared with wild-type mice (Hölscher et al., 2008). The controversial results might be due to differences in Mtb strains used, doses of infection, differences in genetic backgrounds, etc. The detailed description about the *in vivo* role of TLRs in mycobacterial infection is summarized in excellent earlier reviews by Stenger and Modiln (2002); Doherty and Arditi (2004); and Quesniaux et al. (2004). However, the exact roles and regulatory mechanisms by which TLRs affect innate immunity and antimicrobial responses have not been fully characterized.

**Figure 1:**
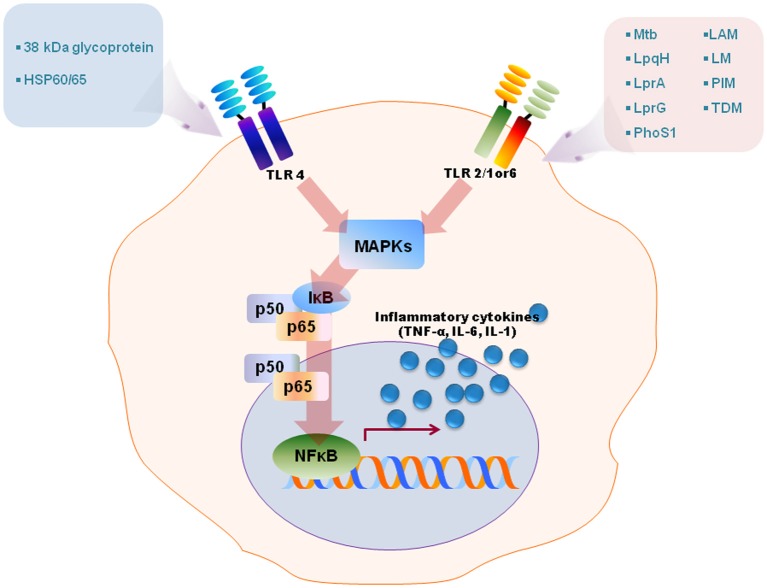
**A schematic model for TLRs activation by diverse mycobacterial antigens.** TLRs are involved in the innate recognition and responses in innate immune cells to numerous mycobacterial antigens. Some mycobacterial antigens including LpqH, lipoarabinomannan (LAM), lipomannan (LM), 38-kDa antigen, LprG, LprA, PhoS1, trehalose dimycolate (TDM), phosphatidylinositol mannoside (PIM) activate TLR2/1 or 6, whereas TLR4 recognizes heat shock protein (HSP) 60/65 and 38-kDa antigen. TLR activation by mycobacterial antigens leads an intracellular signaling pathway that culminates in the production of proinflammatory in macrophages and dendritic cells through MAPK and NF-κB pathways.

Numerous studies have revealed that TLR2 is involved in the innate recognition and responses in innate immune cells such as macrophages and dendritic cells of a variety of mycobacterial cell wall antigens including 19-kDa mycobacterial lipoprotein, glycolipids like lipoarabinomannan (LAM), LM, 38-kDa antigen, LprG lipoprotein, phosphatidylinositol mannoside (PIM), triacylated (TLR2/TLR1), or diacylated (TLR2/TLR6) lipoproteins [reviewed in Jo et al. (2007); Kleinnijenhuis et al. (2011)]. TLR4 is activated by heat shock protein 60/65 and 38-kDa antigen, whereas TLR9 recognizes unmethylated CpG motifs of mycobacterial DNA [reviewed by Jo et al. (2007); Kleinnijenhuis et al. (2011)]. Stimulation of TLRs by mycobacterial ligands leads to an initiation of intracellular signaling cascades culminating in proinflammatory cytokine generation in macrophages and dendritic cells through activation of NF-κB and MAPK pathways (Jo et al., 2007, summarized in Figure 1). Along with canonical NF-κB and MAPK-dependent signaling, it may also be noted that the transcription factor nuclear factor of activated T cells 5 (NFAT5) can be activated by TLR signaling induced by Mtb stimulation, and the co-infection of HIV-1 in tuberculosis accelerates an increase of viral load through expression of NFAT5 (Ranjbar et al., 2012). Recent reports have highlighted that other cells such as alveolar epithelial cells play an important role in innate defense to produce the chemokine CCL2 and also in the pathogenesis of mycobacterial infection (Chuquimia et al., 2012). Additionally, mast cells are activated by mycobacterial LAM to induce the generation of cysteinyl leukotriene through TLR2-NF-κB-dependent signaling (Bąbolewska et al., 2012). Together, these studies suggest that TLRs have a crucial role in the host immune responses against mycobacterial infection and that they are involved in not only immune cells but also other cells. These findings have been summarized in Figure 1. In this context, it is pertinent to note that while, to the best of our knowledge, there is no detailed report on the temporal dynamics of NF-κB activation during infection, our (Basu laboratory) unpublished observations from *ex vivo* experiments using Mtb-infected macrophages, indicate that the upregulation of NF-κB dampening molecules such as TNFAIP3 (A20) occurs as early as 24 h post-infection. It is obvious that Mtb elicits inflammation-dampening signals in macrophages which would favor its survival.

## TLRs signaling by mycobacterial components

Many previous studies have reported that mycobacterial components are involved in innate recognition and responses through TLR signaling (Basu et al., 2007; Pathak et al., 2009; Bansal et al., 2010, 2011; Heo et al., 2011; Byun et al., 2012a,b). Antigens of the PE_PGRS family, namely, PE_PGRS 17 (Rv0978c) and PE_PGRS 11 (Rv0754), recognize TLR2 to induce maturation and activation of human dendritic cells, and enhance the ability of dendritic cells to stimulate CD4(+) T cells (Bansal et al., 2010). These PE_PGRS proteins activate dendritic cells through ERK1/2 and p38 MAPK signaling pathways. Basu et al. have shown that PE_PGRS 33 signals through TLR2 to release TNF-α to induce apoptosis of macrophages (Basu et al., 2007).

**Figure 2:**
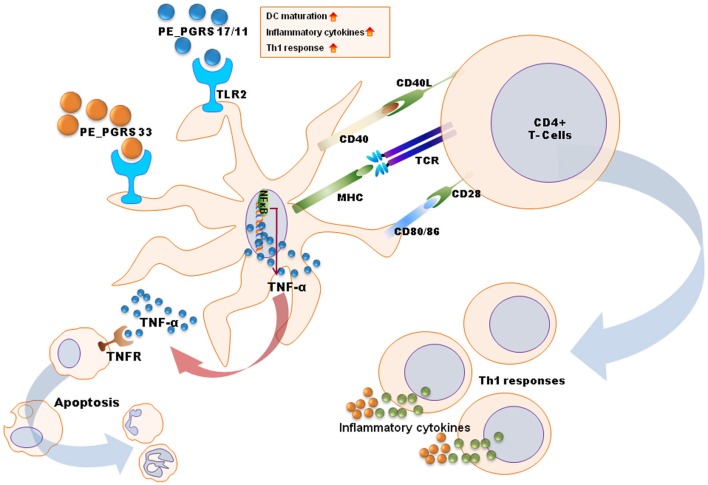
**A schematic diagram for mechanisms of dendritic cell maturation/activation to stimulate T cells by mycobacterial antigens.** PE_PGRS proteins activate dendritic cells through TLR2-MAPK-NF-κB signaling pathways. PE_PGRS 33-induced TNF-α by dendritic cells induces apoptosis in macrophages. Others (e.g., Rv0462, Rv0315, Rv0577, and PE_PGRS 17/11) increase expression of costimulatory molecules (CD80, CD86, and class II MHC) and proinflammatory cytokines (TNF-α, IL-1β, IL-6, and IL-12), leading to Th1 immune responses.

Other Mtb proteins have been shown to influence dendritic cell function. Rv0462 (lipoamide dehydrogenase C), Rv0315, and Rv0577 (Mtb-restricted secretory protein involved in the methylglyoxal detoxification pathway) induce dendritic cell maturation and activation leading to increased expression of costimulatory molecules (CD80, CD86, and class II MHC) and proinflammatory cytokines (TNF-α, IL-1β, IL-6, and IL-12), and potentiate the Th1 immune response (Heo et al., 2011; Byun et al., 2012a,b). These findings have been summarized in Figure 2.

Yet another effecter of proinflammatory cytokine production is trehalose dimycolate (TDM). TDM has been reported to be tethered to several receptors, including TLR2, the class A scavenger receptor MARCO, Fc receptor-γ (FcRγ), and macrophage-inducible C-type lectin (Mincle) (Bowdish et al., 2009; Ishikawa et al., 2009; Werninghaus et al., 2009). TDM triggers MARCO/TLR2/CD14-dependent signaling to produce proinflammatory cytokines (Bowdish et al., 2009). In addition to TLR2-dependent signaling, TDMs also activate macrophages and dendritic cells via FcRγ-Syk-Card9 pathway (Werninghaus et al., 2009). Mincle, which is one of the C-type lectin receptors expressed in macrophages subjected to several types of stress (Matsumoto et al., 1999), is an essential receptor for TDM-dependent inflammatory responses, nitric oxide synthesis, and granuloma formation (Ishikawa et al., 2009). TDM receptors are associated with partial protection against Mtb infection (Bowdish et al., 2009; Ishikawa et al., 2009; Werninghaus et al., 2009).

Recent work by Bansal et al. (2011) suggests that upon infection with *M. bovis* bacille Calmette-Guérin (BCG), TLR2 signaling activates Wnt-β-catenin signaling. The authors suggest that TLR2 integrates Wnt-β-catenin signaling to modulate a battery of genes associated with T(Reg) cell lineage commitment. At low MOIs, Mtb-triggered macrophage apoptosis depends on a TLR2 signaling cascade associated with ASK1/p38 MAPK- and c-Abl-dependent phosphorylation of c-FLIP and its degradation, leading to caspase 8 activation (Kundu et al., 2009).

TLR-dependent NF-κB signaling and MAPK pathways contribute to antimycobacterial innate immunity through secretion of antibacterial effector molecules, cytokines, and chemokines, thus recruiting various immune cells to the site of infection (Jo et al., 2007; Jo, 2010; Huynh et al., 2011; Kleinnijenhuis et al., 2011). In contrast, Mtb-mediated TLR2 signaling is associated with generation of an environment favoring the survival of Mtb in macrophages (Yoshida et al., 2009). Together, TLR signaling in mycobacterial infection acts as a double-edged sword regulating a delicate balance in host innate and inflammatory responses to determine disease outcome.

## Human TLR polymorphism and mycobacterial infection

Human genetic polymorphisms of TLRs have a role in regulating innate recognition of microbes and in determining susceptibility to mycobacterial infection. The relationship between TLR polymorphisms, disease susceptibility, and innate immune activities against mycobacteria has been described in several reviews (Texereau et al., 2005; Lykouras et al., 2008; Kleinnijenhuis et al., 2011). Here we describe the recent data on genetic and functional studies of TLRs in protection and pathogenesis of mycobacterial infection. It has been reported that TLR1/6-deficient genotypes (typified by TLR1_T1805G and TLR6_C745T), after vaccination with *M. bovis* BCG, are linked with the increased production of Th1-type T cell cytokines through regulation of monocyte IL-10 production (Randhawa et al., 2011). Other recent studies have reported that rs352139, an SNP located in the intron of TLR9 is associated with tuberculosis susceptibility in Indonesian and Vietnamese populations (Kobayashi et al., 2012). It has also been reported that TLR8 polymorphisms are associated with pulmonary tuberculosis susceptibility in males (Davila et al., 2008). Interestingly, TLR8 transcriptional levels are increased in patients at the early phase of infection and TLR8 protein expression is increased in macrophages after *M. bovis* BCG infection (Davila et al., 2008).

Previously, it was shown that human TLR1 deficiency is linked with impaired mycobacterial innate immune signaling and susceptibility to leprosy (Misch et al., 2008). Recent studies have shown that TLR chaperones, PRAT4A, and PRAT4B are important regulators in TLR1 trafficking to the cell surface and are also regulated by IFN-γ (Hart and Tappiung, 2012). Furthermore, there is an inverse correlation between Euro-American lineage of Mtb and extra-pulmonary dissemination, suggesting that a link between interaction between host and mycobacterial genotypes and the clinical progression of tuberculosis (Caws et al., 2008). Future studies are warranted to define the exact relationship between host and bacterial genotype and disease phenotype using clinical isolates.

## Cross talk between TLR, autophagy, and vitamin D signaling pathways

Several other innate immune pathways are closely linked with the TLR signaling pathway, thus cooperatively defining the innate immune response which enables clearance of mycobacteria inside cells. Activation of autophagy has a critical effector role in innate immune responses such as enhancement of phagosomal maturation and coordination of the innate and adaptive immune systems (Deretic, 2009, 2012). It is known that a variety of pathogen- or danger-associated molecular patterns and pattern-recognition receptor signaling are closely associated with autophagy activation (Xu et al., 2007; Delgado et al., 2008; Fabri et al., 2011a; Deretic, 2012). Therefore, it is not surprising that TLR signaling pathways triggered by engagement of mycobacterial antigens lead to activation of autophagy (Shin et al., 2010). The activation of autophagy influences the antigen-presenting capacity of the immunodominant mycobacterial antigen Ag85B by antigen-presenting cells (Jagannath et al., 2009), as well as the production of antimicrobial proteins and pro-inflammatory cytokines (Yuk et al., 2009; Shin et al., 2010), thereby influencing vaccine efficacy and host immune defense during mycobacterial infection.

Recent studies have uncovered the interplay of vitamin D-dependent antimicrobial responses and autophagy pathways in the activation of host defense against mycobacterial infection (Yuk et al., 2009; Jo, 2010; Shin et al., 2010; Fabri et al., 2011a). Activation of TLR signaling in human monocytes/macrophages has been shown to lead to the induction of the antimicrobial peptide cathelicidin, which is critically involved in antimicrobial responses against Mtb (Liu et al., 2006, 2007; Yang et al., 2009). Physiological concentrations of 1,25D3 were found to be enough to induce the production of the human cathelicidin and autophagic flux, thereby activating antimicrobial activities against Mtb and also inhibiting HIV replication in cells co-infected with Mtb and HIV (Yuk et al., 2009; Campbell and Spector, 2012). Moreover, cathelicidin plays a dual role in vitamin D-induced autophagy activation in human monocytes/macrophages, acting both as a critical effector of antimicrobial responses against Mtb and as a mediator of the autophagy pathway through enhancement of autophagic flux and the induction of autophagy-related genes (Yuk et al., 2009; Campbell and Spector, 2012). Importantly, in human macrophages cultured with vitamin D-sufficient sera, IFN-γ production by Th1 cells as well as stimulation of TLR2/1, turn on antimicrobial activity through antimicrobial peptide expression, autophagy activation, and phagosome-lysosome fusion (Fabri et al., 2011b). These findings provide insights into how vitamin D-dependent signaling triggers antimicrobial activity by activating autophagy and regulating both innate and adaptive immune responses.

The impact of host autophagy on phagosome maturation and Mtb killing gives rise to the idea that compounds that activate or modulate the autophagy pathway, could potentially enhance antimicrobial activities against Mtb infection (Gutierrez et al., 2004; Singh et al., 2006; Fabri et al., 2011a; Kim et al., 2012; Lam et al., 2012; Zullo and Lee, 2012). Recent studies have shown that the prevalent anti-TB drugs isoniazid and pyrazinamide promote autophagy activation. This plays an important role in successful antimicrobial responses during therapy against mycobacterial infection *in vivo* and *in vitro* (Kim et al., 2012). Lam et al. also showed that the antiprotozoal drug nitazoxanide stimulates autophagy induction and mTORC1 inhibition, and inhibits Mtb proliferation in human monocytes and monocytic cells (Lam et al., 2012).

## Concluding remarks

Developing vaccines and chemotherapeutic agents is the cornerstone of the successful management of tuberculosis. Understanding the molecular mechanisms of specific interactions of Mtb with its host should augment efforts in both these areas. Immune modulation as a strategy to combat mycobacterial infection remains underexplored. This review brings to light how mycobacterial modulins signaling through TLRs, contribute either to an effective host immune response or to immune evasion by the pathogen. Targeting those modulins that facilitate immune evasion, or exploiting those that facilitate a robust innate and adaptive immune response, could offer new avenues for controlling infection. The review also brings forth the attractive possibility of developing therapeutics designed to augment autophagy as a means of restricting mycobacterial survival and combating infection.

### Conflict of interest statement

The authors declare that the research was conducted in the absence of any commercial or financial relationships that could be construed as a potential conflict of interest.
